# Leaf morphological and anatomical traits from tropical to temperate coniferous forests: Mechanisms and influencing factors

**DOI:** 10.1038/srep19703

**Published:** 2016-01-22

**Authors:** Miao Tian, Guirui Yu, Nianpeng He, Jihua Hou

**Affiliations:** 1 Key Laboratory of Ecosystem Network Observation and Modeling, Institute of Geographic Sciences and Natural Resources Research, Chinese Academy of Sciences, Beijing 100101, China; 2 The Key Laboratory for Forest Resources & Ecosystem Processes of Beijing, Beijing Forestry University, Beijing 100083, China

## Abstract

Leaf traits may reflect the adaptation mechanisms of plants to the environment. In this study, we investigated leaf morphological and anatomical traits in nine cold-temperate to tropical forests along a 4,200-km transect to test how they vary across latitudinal gradients. The results showed that leaf dry weight decreased (*P* < 0.05), while specific leaf area (SLA) increased (*P* < 0.05) with increasing latitude. Stomatal length and stomatal density did not change significantly, while stomatal pore area index increased (*P* < 0.05) with increasing latitude. The palisade-leaf mesophyll thickness ratio increased (*P* < 0.01), while the spongy-leaf mesophyll thickness ratio decreased, with increasing latitude (*P* < 0.01). Climate and leaf nutrients were the main factors that regulated leaf morphological and anatomical traits. Furthermore, we identified positive correlations between leaf area and leaf dry weight, leaf thickness and palisade mesophyll thickness, but negative correlations between stomatal length and stomatal density (all *P* < 0.01). The observed negative correlations represented the adaptive mechanisms of leaves through their morphological and anatomical traits. These findings provided new insights into the responses of leaf morphological and anatomical traits to climate changes and important parameters for future model optimization.

Leaves play key roles in plant function and long-term adaptation to the environment. Although comprising basically of epidermis, stomata, and mesophyll, leaves exhibit apparent differences in area, thickness, and shape among different species, as a result of phylogenetic relationships and adaptation to specific environments^1^. Some studies have investigated how morphological traits of the leaf economic spectrum, such as leaf area, and specific leaf area, vary across large geographical scales and ecosystems and adapt to environmental factors^2^,^3^. However, it remains unclear whether variations in leaf anatomical traits are associated with plant adaptation to different environments across a large geographical scale.

It is known that leaf area and SLA may reflect plant photosynthetic capacity on large geographical scales^4^,^5^. Relatively high leaf area and SLA may enhance plant photosynthetic capacity and primary productivity^6^. Some studies have demonstrated that SLA is negatively correlated to leaf life span at species level^7^,^8^,^9^; however, it is still unknown whether SLA increases with the decreasing leaf lifespan on the large geographical scale. Furthermore, leaf morphological traits may better reflect the changes in environmental factors such as temperature^10^,^11^,^12^, light intensity^13^, and water status^14^.

Stomata are microscopic structures on the epidermis of leaves bounded by a pair of guard cells, that control water vapor and gas exchange between plants and atmosphere^15^. In response to changing environmental conditions, leaves can open or close, or exhibit long-term adaptations of stomatal morphology^16^. Previous studies have shown that stomatal density is negatively correlated to stomatal size or length^17^, and that stomatal characteristics are susceptible to environmental changes^5^,^18^,^19^,^20^, such as light intensity^21^, temperature^22^,^23^,^24^, and water status^25^. However, it is important to investigate whether the adaptation strategies of stomata observed in short-time growth experiments are applicable to natural ecosystems, in order to provide an efficient approach for assessing plant photosynthesis and transpiration on a large geographical scale.

Photosynthesis occurs in the chloroplasts of palisade and spongy tissues in the mesophyll. Spongy tissues have larger inter cellular space for gas transportation, while palisade tissue is beneficial to increase leaf photosynthesis^26^,^27^. Previous studies paid little attention to the adjustment of internal structure in the mesophyll. Additionally, the anatomical structure of leaves is regulated by many environmental factors such as temperature, water status^11^, and light intensity^28^,^29^. Wang *et al*.^5^ found that a thicker leaf blade may increase leaf water content under dry conditions. However, the correlation of leaf structure and adaptive mechanisms in natural ecosystems has not been verified on a large geographical scale.

It is known that the leaf economic spectrum refers to leaf life and physiology, and includes two strategies of resource utilization. At the quick-return end, leaves have a high photosynthetic rate, short leaf lifespan, and low-cost dry-mass investment, while at the slow-return end, leaves present a reverse trend and long leaf lifespan^4^. We hypothesized that species with a short leaf lifespan at high latitude may choose the quick-return end, in order to achieve high photosynthetic rates during the short growing period. However, it remains unclear whether this adaptation strategy reflects to any leaf anatomical traits.

In order to explore the changes in leaf traits and the underlying adaptation mechanisms on a large geographical scale, we conducted an integrative investigation of leaf morphological and anatomical traits, including leaf area, leaf dry weight, SLA, stomatal length, stomatal density, stomatal pore area index (SPI), leaf thickness, palisade-leaf mesophyll thickness ratio, and spongy-leaf mesophyll thickness ratio, in 99 tree species from nine tropical to temperate coniferous forests, in Huzhong (HZ), Liangshui (LS), Changbai (CB), Dongling (DL), Taiyue (TY), Shennong (SN), Jiulian (JL), Dinghu (DH), and Jiangfeng (JF), along a 4,200-km transect (Fig. 1; Tables 1 and 2). The main objectives of this study were to 1) investigate how leaf morphological and anatomical traits change with latitudinal gradients, temperature, and precipitation; 2) explore the underlying adaptation mechanisms (trade-off among different traits) of leaf traits to environmental pressure; and 3) understand the main factors regulating leaf morphological and anatomical traits on a large scale. This study aims to provide a basis for understanding plant responses to climate change.

## Results

### Changes in leaf morphological and anatomical traits among forests

Across all species, the mean leaf area was 21.81 cm^2^ per individual, leaf dry weight was 164.15 mg per individual, and SLA was 13.65 mm^2^ mg^−1^. The range of leaf area was 0.04–157.78 cm^2^ per individual, of leaf dry weight was 0.40–582.83 mg per individual, and of SLA was 1.89–47.63 mm^2^ mg^−1^ (Fig. S1). Leaf area, leaf dry weight, and SLA differed significantly among different forests (all *P* < 0.01; Table 3).

Additionally, the mean stomatal length was 12.99 μm, the mean stomatal density was 256.50 individual mm^−2^, and the mean SPI was 3.61% (Fig. S1). The range of stomatal length was 4.29–36.22 μm, of stomatal density was 14.73–840.77 individual mm^−2^, and of SPI was 0.46–19.72% (Fig. S1). Stomatal length differed significantly among different forests (*P* < 0.01; Table 3). Moreover, the mean of leaf thickness was 136.14 μm, of palisade-leaf mesophyll thickness ratio was 33.64%, and the mean spongy-leaf mesophyll thickness ratio was 43.16% (Fig. S1). The range of leaf thickness was 39.99–521.46 μm, of the palisade-leaf mesophyll thickness ratio was 14.37–54.32%, and of the spongy-leaf mesophyll thickness ratio was 21.07–73.18% (Table 3). Leaf area, palisade-leaf mesophyll thickness ratio, and spongy-leaf mesophyll thickness ratio differed significantly among different forests (*P* < 0.05). The values of leaf thickness in DL, TY, SN, and JL were significantly lower than those in HZ, LS, CB, DH, and JF (*P* < 0.01). In addition, the palisade-leaf mesophyll thickness ratio in JF was significantly lower than those in HZ, LS, CB, DL, TY, and SN, while the spongy-leaf mesophyll thickness ratio in JF was significantly higher than those in HZ, LS, CB, DL, TY, and SN (*P* < 0.01; Table 3).

### Latitudinal changes in leaf morphological and anatomical traits

Leaf area showed no latitudinal trend (Fig. 2A), while leaf dry weight (R^2^ = 0.56, *P* < 0.05) decreased linearly and SLA (R^2^ = 0.72, *P* < 0.01) increased linearly with increasing latitude (Fig. 2B,C). Stomatal length and stomatal density showed no significant latitudinal changes, while SPI increased significantly with increasing latitude (R^2^ = 0.47, *P* < 0.05; Fig. 2D–F). Leaf thickness, palisade-leaf mesophyll thickness ratio, and spongy-leaf mesophyll thickness ratio all showed significant latitudinal patterns. Leaf thickness and spongy mesophyll thickness first decreased and then increased with increasing latitude (R^2^ = 0.73, *P* < 0.05) (Fig. 2G and Fig. S4B), however, the palisade-leaf mesophyll thickness ratio and the palisade-spongy mesophyll ratio increased (R^2^ = 0.69, *P* < 0.01) with increasing latitude, while the spongy-leaf mesophyll thickness ratio decreased (R^2^ = 0.76, *P* < 0.01) (Fig. 2H,I and Fig. S4C).

### Correlations among leaf morphological and anatomical traits

SLA, SPI, and palisade-leaf mesophyll thickness ratio were negatively correlated with the spongy- leaf mesophyll thickness ratio (*P* < 0.05), while SPI was positively correlated to the palisade-leaf mesophyll thickness ratio (*P* < 0.01; Table S1). Leaf area increased linearly with increasing leaf dry weight (R^2^ = 0.44, *P* < 0.01; Fig. 3A), while stomatal length decreased with increasing stomatal density (R^2^ = 0.27, *P* < 0.01; Fig. 3B). Additionally, leaf thickness increased significantly with increasing palisade mesophyll thickness (R^2^ = 0.69, *P* < 0.01) and spongy mesophyll thickness (R^2^ = 0.83, *P* < 0.01; Fig. 3C,D).

### Main factors regulating leaf morphological and anatomical traits

Leaf dry weight increased and SLA decreased with increasing maximum monthly temperature (*P* < 0.05) (Fig. S2A and B). Furthermore, stomatal length and SPI were negatively correlated (*P* < 0.01) (Fig. 4B and Fig. S2C), and stomatal density was positively correlated to the maximum monthly temperature (*P* < 0.01) (Fig. 4C). In addition, the palisade-leaf mesophyll thickness ratio (R^2^ = 0.69, *P* < 0.01) decreased and the spongy-leaf mesophyll thickness ratio (R^2^ = 0.56, *P* < 0.01) increased with increasing maximum monthly temperature (Fig. S2D and E). No significant correlations were observed between any morphological and anatomical traits and the de Martonne aridity index, except for leaf thickness (R^2^ = 0.52, *P* < 0.05; Fig. 4A).

Structural equation models showed that climate and soil nutrients explained more than 60% of the variations in leaf area (R^2^ = 0.42). Additionally, leaf nutrients had a 78% direct effect on leaf dry weight (R^2^ = 0.72), and the climate had a 69% direct effect on SLA (Table 3). More than 50% of the variation in stomatal length (R^2^ = 0.37) could be explained by climate and leaf nutrients, while the climate had a more than 30% direct effect on stomatal density (R^2^ = 0.11). However, climate, leaf nutrients, and soil nutrients could explain only a small proportion of the variation in SPI (Table 3). Soil nutrients had a 50% direct effect on leaf thickness (R^2^ = 0.27), while, a high proportion of the variation in the palisade-leaf mesophyll thickness ratio (R^2^ = 0.24) and the spongy-leaf mesophyll thickness ratio (R^2^ = 0.29) could be explained by the climate (Table 3).

## Discussion

### Latitudinal patterns and adaptive strategies of leaf morphology traits

Leaf dry weight and SLA showed significant latitudinal patterns from tropical to temperate coniferous forests along a 4,200-km transect, but there was no clear spatial pattern for leaf area (Fig. 2). Previous studies have reported that leaf area has not apparent trend with the changing environmental factors^30^,^31^,^32^. It is widely recognized that a large leaf area can enhance solar energy capture^22^, but also increases evapotranspiration. Therefore, leaf area is controlled in such way to keep the nutrient content at an optimal level for the given light and water status^33^.

Leaf dry weight decreased significantly with increasing latitude, which reflected the leaf construction investment (Fig. 2B). SLA is an integrative parameter of leaf area and leaf dry weight that increased with increasing latitude (Fig. 2C), indicating a higher photosynthesis capacity per unit of leaf dry biomass in higher latitudes. The results were consistent with the assumption that leaf lifespan is negatively correlated to SLA^4^,^34^, since leaf lifespan is shorter in higher latitude regions. Therefore, the increasing SLA with latitude may be one of the adaptive strategies of leaf morphological traits to the changing environment in order to maximize the photosynthetic rate at higher latitude regions.

### Latitudinal patterns and adaptive strategy of leaf anatomical traits

Stomatal length and stomatal density varied slightly from tropical to temperate coniferous forests, but SPI significantly increased with increasing latitude (Fig. 2). Stomatal length decreased and stomatal density increased with increasing maximum monthly temperature (Fig. 4B,C). A previous study demonstrated that cell differentiation increases under relatively higher temperature conditions, resulting in increased stomatal density^35^. SPI is an integrative parameter of stomatal density and stomatal length that reflects the stomatal conductance of leaves and increased SPI leads to higher stomatal conductance and photosynthetic capacity in leaves^36^. Therefore, a higher SPI in higher latitudinal regions may maximize carbon gain and increase plant growth during a relatively short growing season. Therefore, increasing SPI maximizes the photosynthetic rate at higher latitude and is one of the adaptive strategies of leaf stomatal traits to the changing environment.

Stomatal traits and open-close behaviors partially determine the balance of CO_2_ uptake for photosynthesis against water loss by transpiration^15^. For different plant species and environmental conditions, the range of leaf stomatal length is 4.29–36.22 μm and density 14.73–840.77 individual mm^−2^. Despite the large variability in stomatal length and density, these variables have a negative relationship^15^,^17^. Small stomata can open and close more rapidly, and in high densities, they allow the rapid increase in stomatal conductance that maximizes CO_2_ diffusion for photosynthesis under favorable environmental conditions^15^. Our findings supported that stomatal length was negatively correlated to stomatal density on a large geographical scale (Fig. 3B).

Leaf thickness was positively correlated to de Martonne aridity index (Fig. 4A), indicating that drought conditions are not suitable for leaf growth. The palisade-leaf mesophyll thickness ratio reflects the proportion of palisade mesophyll thickness and leaf thickness, and a relatively high ratio indicates the high amount of palisade tissue in the leaf. Previous studies have demonstrated that the number of palisade parenchyma cells is positively associated with the amount of chlorenchyma and thus the photosynthetic capacity^31^. Therefore, a higher palisade-leaf mesophyll thickness ratio in high latitudes may enhance the photosynthetic capacity during a short growing season. The spongy-leaf thickness mesophyll ratio reflects the ratio of spongy mesophyll thickness and leaf thickness, and a relatively high ratio indicates the higher amount of spongy tissue. Forest canopy density increases with decreasing latitude and increasing complexity of ecosystem structure; thus, scattered light increases with decreasing latitude. Spongy mesophyll can absorb a higher scattering of light intensity and increase the light absorption at low light intensities^37^. Therefore, plant species with a higher spongy-leaf mesophyll thickness ratio in low latitudes may better utilize scattered light. Overall, these strong relationships between anatomical traits reflect the adaptation of plants to changing environmental conditions by regulating the ratios of leaf anatomical structure.

### Leaf anatomical traits and the leaf economic spectrum

Our results showed that leaf morphological and anatomical traits might reflect the trade-off mechanism of leaf resource investment and optimal leaf photosynthesis capacity. Leaves with a short lifespan have lower leaf dry weight, but higher SLA, SPI, and palisade-leaf mesophyll thickness ratio, when vegetation changes from evergreen broad-leaved forests toward deciduous coniferous forests with increasing latitude. It seems that leaves tend to choose the quick-return strategy with increasing latitude. Leaves increase their photosynthetic rate and reduce their construction investment with increasing latitude, which may help them to produce more photosynthetic products during a short growing period. However, leaves with a long lifespan have higher leaf dry weight, but lower SLA, SPI and palisade-leaf mesophyll thickness ratio with decreasing latitude, suggesting that the leaf decreases the photosynthetic rate and increases leaf construction with decreasing latitude, in order to maintain a longer leaf lifespan. Consistently, leaves tend to choose a slow-return strategy with decreasing latitude^4^.

The main factors regulating the leaf economic spectrum are still being debated. Our results showed that the regulating factors differ between leaf morphological and anatomical traits, suggesting that anatomical traits play a significant role in the leaf economic spectrum. Leaf area is mainly influenced by climate and soil nutrients (>50%), leaf dry weight by leaf nutrients (>70%), and SLA by the climate (>60%) (Table 3). Leaf nutrients and climate play important roles in stomatal traits (Table 4). Leaf thickness is controlled by soil nutrients, but the palisade-leaf mesophyll thickness ratio and spongy-leaf mesophyll thickness ratio are influenced by the climate (Table 4). The palisade-leaf mesophyll thickness ratio is decreased, while the spongy-leaf mesophyll thickness ratio is increased with increasing maximum monthly temperature (Fig. S2D and E). A plausible explanation might be that temperature influences plant metabolic and growth rates by regulating plant lifespan^12^. Overall, it is required to expand the leaf economic spectrum theory through an integrative study of leaf morphological and anatomical traits that will allow us to better understand the resource utilization and the adaptation strategies to changing environments.

## Conclusion

To our knowledge, this is the first study that combined leaf morphological and anatomical traits to explore the adaptation strategies and resource investment strategies of plants on a large geographical scale. SLA, SPI, and the palisade-leaf thickness mesophyll ratio increased with increasing latitude, while leaf dry weight and the spongy-leaf thickness mesophyll ratio decreased with increasing latitude. The strong correlations of leaf dry weight to leaf area, palisade mesophyll thickness to leaf thickness, and spongy mesophyll thickness to leaf thickness reflected the adaptive strategies of leaf morphological and anatomical traits. Furthermore, the regulation of stomatal length and density by a trade-off mechanism that leads to an increased SPI with increasing latitude, was probably an adaptive strategy. A relatively higher SLA, palisade-leaf mesophyll thickness ratio, and spongy-leaf mesophyll thickness ratio enhanced photosynthetic efficiency at high latitudes, while the high-cost leaf construction investment was important to maintain long leaf lifespan at low latitudes. Furthermore, the factors that regulated leaf morphological and anatomical traits were different, and the explanation of the leaf economic spectrum became even more complicated. These results provided new insights into the adaptive strategies of plants at the morphological and anatomical level.

## Materials and Methods

### Site description

**Figure 1:**
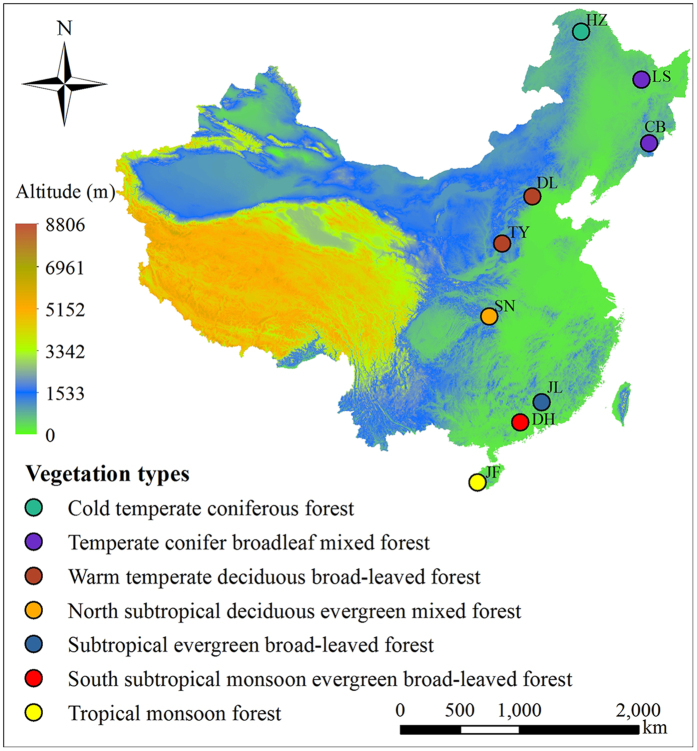
Geographic locations of nine randomly selected forests along the north-south transect of eastern China. HZ: Huzhong; LS: Liangshui; CB: Changbai; DL: Dongling; TY: Taiyue; SN: Shennong; JL: Jiulian; DH: Dinghu; JF: Jianfeng. The figure was created by Miao Tian using Arcgis 9.2 (ESRI, USA).

The north-south transect of eastern China (NSTEC) is a unique forest belt mainly driven by thermal gradients, and encompasses almost all forest types found in the northern hemisphere. Nine forests along the NSTEC were selected for field sampling as shown in Fig. 1. The latitude range of these forests is between 18.74° and 51.78° (>4,200-km), the mean annual temperature (MAT) ranges between –4.40 and 19.80 °C, and the annual precipitation (MAP) ranges between 481.60 and 2,449.00 mm. Detailed information about the sampling sites is provided in Table 1. In each forests, we randomly selected sampling sites within the national nature reserves, in order to avoid anthropogenic disturbances.

### Field sampling

Field sampling was conducted during July and August in 2013. Four experimental plots (30 × 40 m) were set up in each forest ecosystem. Geographic information (latitude, longitude, and altitude), plant species composition, and community structure were investigated for each plot. Data on the number, height, diameter at breast height (≥2 cm) of all trees (basal stem diameter for shrubs and coverage for herbs), and other important traits were collected. We collected leaf samples from 10−13 dominant tree species in each plot (99 tree species in total) based on the importance value that was calculated from the relative density, relative frequency, and total relative dominance^38^ of selected tree species. The total importance value of these selected plant species ranged between 43% and 99% across the forests (Table 1).

A total of 20 fully expanded sun leaves were collected from four individuals of each species, and all the leaf samples from each plot were bulked together representing one replicate^39^. Leaf samples were immediately stored in a cool box with ice and transported to the lab. Soil samples from each plot were randomly collected from the 0–10 cm layer using a soil sampler (6 cm in diameter).

### Measurement of leaf traits

Here, we studied three leaf morphological traits including leaf area, leaf dry weight, and specific leaf area (SLA) that reflect the strategies of leaf construction, investment, and photosynthesis, respectively and also six leaf anatomical traits, including stomatal length, stomatal density, stomatal pore area index (SPI), leaf thickness, palisade-leaf mesophyll thickness ratio, and spongy-leaf mesophyll thickness ratio that reflect the long-term adaptation of leaf stomatal morphology and photosynthetic capacity (Table 2).

### Measurement of leaf morphological traits

Leaf area (cm^2^ per individual) was measured using a scanner (Cano Scan LIDE 110, Japan) and Photoshop CS (Adobe, USA). Then, five leaves were dried in a dryer at 60 °C to obtain leaf dry weight (mg per individual). SLA (mm^2^ mg^−1^), or leaf area per unit of dry mass, was calculated as Eq. 1^7^.


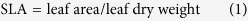


### Measurement of leaf anatomical traits

**Figure 2:**
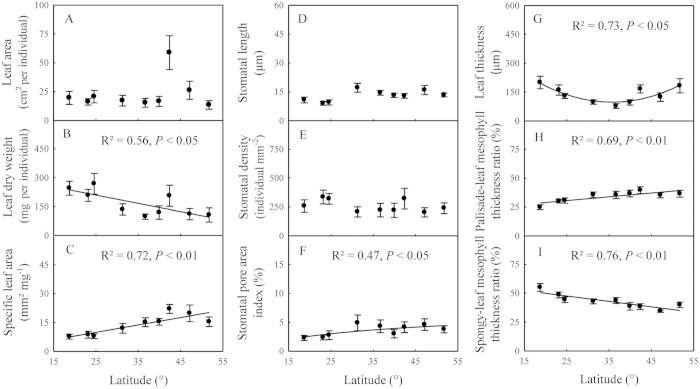
Latitudinal trends of leaf morphological (**A–C**) and anatomical (**D–I**) traits.Data are presented as means ± standard errors.

**Figure 3:**
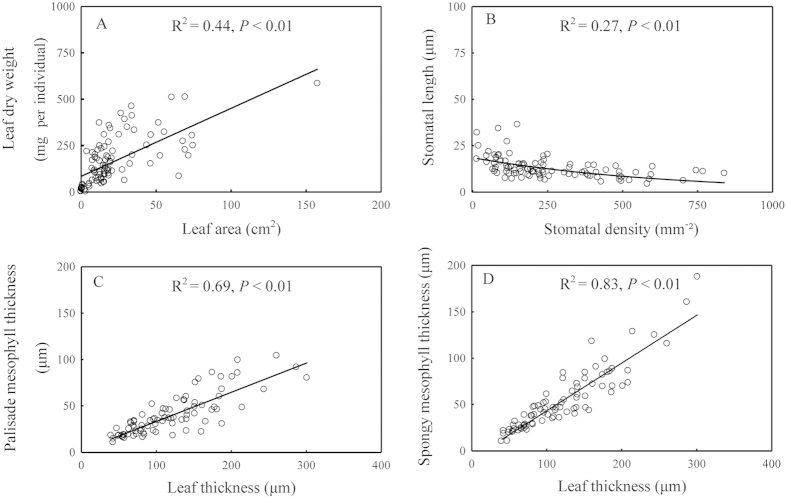
Correlations between leaf morphological traits (**A**) and leaf anatomical traits (**B–D**).

**Figure 4:**
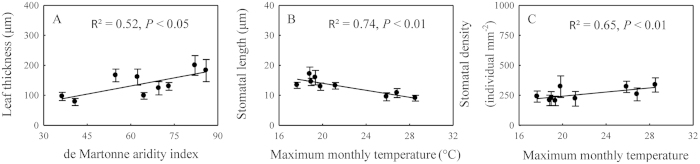
Changes in leaf thickness, stomatal length, and stomatal density with climate parameters. Bars show standard errors.

In order to measure leaf anatomical traits, rectangular pieces (1 cm × 0.5 cm) that included the midrib and a portion of the lamina were cut from the leaves (Fig. 2) and fixed in formalin-acetic acid- alcohol solution (FAA, 5 ml of 38% formalin, 5 ml of glacial acetic acid, and 90 ml of 50% ethanol with 5 ml glycerin)^40^,^41^. Three pre-treated leaves from each species were randomly selected to measure leaf anatomical traits. Stomatal traits were measured avoiding leaf veins (Fig. S3). Leaf samples were dried in a fume cupboard, and two fields were photographed using a scanning electron microscope (Hitachi, Japan). In each image, we measured stomatal length (μm) via five randomly selected stomata. For the measurement of stomatal density (individual mm^−2^), the number of stomata per unit area (mm^−2^) was counted from the images at a magnification of 320 × (visual field area = 0.112 mm^2^). A total of 30 data points for stomatal length and six data points for stomatal density in each species were obtained. Stomatal length and density were measured using MIPS (Optical Instrument Co., Ltd., Chongqing, China), while SPI (%) was calculated as Eq. 2^36^:


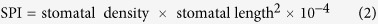


Leaf samples were progressively dehydrated in an ethanol series (50–100%) and infiltrated with warm paraffin (56–58 °C). Leaf samples of 8–12 μm in size were obtained with a rotary microtome (Leica, RM2255, Germany). The slides were stained with safranin and fast green (1% aqueous safranin and 0.5% fast green in 95% ethanol). Then, all sections were conducted at 400 × magnification with light microscope (Leica, DM2500, Germany) to measure leaf thickness (μm), palisade mesophyll thickness (μm) and spongy mesophyll thickness (μm) (Fig. S3). A total of 30 data points of leaf thickness, palisade mesophyll thickness, and spongy mesophyll thickness for each species were measured. Finally, the palisade-leaf mesophyll thickness ratio and the spongy-leaf mesophyll thickness were calculated as Eq. 3 and Eq. 4, respectively^42^.


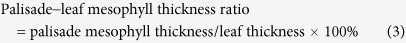



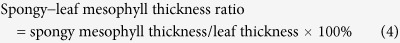


### Other parameters

Fresh soil samples were sieved through a 2 mm mesh to remove roots and visible organic debris. The carbon (C) and nitrogen (N) contents in soil samples were determined by dry combustion using a Vario MAX CN Elemental Analyzer (Elementar, Germany)^43^.

Climate data from 1961 to 2010 at a 1 km × 1 km spatial resolution were obtained from 756 climate stations of the China Meteorological Administration and analyzed using ANUSPLIN^44^. Data on climatic parameters including MAT, MAP, and maximum monthly temperature were collected from the meteorological database based on latitude and longitude. The de Martonne aridity index (DI) was calculated as Eq. 5 to describe the effect of water availability^45^:


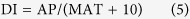


### Data analysis

Leaf traits were log_10_ transformed prior to analysis in order to obtain approximate normality. The relationships between leaf traits and latitude, de Martonne aridity index, and maximum monthly temperature were explored using regression analysis. Models with higher coefficient of determination (R^2^) were chosen as the best-fit models. Structural equation modeling was used to evaluate the effects of climate factors (maximum monthly temperature and MAP), soil nutrients (soil total C and N contents), and leaf nutrients (leaf C and N contents) on leaf traits. Structural equation modeling was used to combine the roles of multiple variables in a single analysis, distinguishing the direct effects from the indirect effects. We examined model fitness using the root mean square error of approximation and the goodness-of-fit index.

Chinese forest maps were produced using ArcMap (9.2, ESRI, USA). Regression analysis, and differences in leaf traits among different forests, and relationships between leaf morphological and anatomical traits were analyzed using SPSS 13.0 (Chicago, IBM Corp., USA).

## Additional Information

**How to cite this article**: Tian, M. *et al*. Leaf morphological and anatomical traits from tropical to temperate coniferous forests: Mechanisms and influencing factors. *Sci. Rep.* **6**, 19703; doi: 10.1038/srep19703 (2016).

## Supplementary Material

Supplementary Information

Supplementary Information

## Figures and Tables

**Table 1:** Basic information of nine randomly selected forests along the north-south transect of eastern China.

Site	Latitude (°)	Longitude (°)	MAT‡ (°C)	MAP (mm)	Maximum monthly temperature (°C)	de Martonne aridity index	Soil carbon content (%)	Soil nitrogen content (%)	Forest types	Dominant trees	No. species	Important value(%)
HZ†	51.78	123.02	−4.40	481.60	17.67	86.00	4.94	0.31	Cold temperate coniferous forest	*Larix gmelinii Betula platyphylla*	11	99§
LS	47.19	128.90	−0.30	676.00	19.35	69.69	7.70	0.46	Temperate conifer broad leaf mixed forest	*Pinus koraiensis Subgen tegmentosum*	11	91
CB	42.40	128.09	2.60	691.00	19.82	54.84	7.04	0.64	Temperate conifer broad leaf mixed forest	*P. koraiensis, Quercus mongolica*	10	88
DL	39.96	115.42	4.80	539.10	21.19	36.42	3.89	0.31	Warm temperate deciduous broad-leaved forest	*Q.mongolica Betula dahurica*	12	91
TY	36.70	112.08	6.20	662.00	18.96	40.86	4.51	0.26	Warm temperate deciduous broad-leaved forest	*P. tabuliformis, Q.wutaishanica*	10	96
SN	31.32	110.50	10.60	1330.00	18.83	64.56	4.19	0.38	North subtropical deciduous evergreen mixed forest	*Fagaceae Comaceae*	13	59
JL	24.58	114.44	16.70	1954.00	25.89	73.18	3.57	0.23	Subtropical evergreen broad-leaved forest	*Fagaceae Ebenaceae*	10	51
DH	23.17	112.54	20.90	1927.00	28.53	62.36	2.81	0.18	South subtropical monsoon broad-leaved forest	*Theaceae Ebenaceae*	11	77
JF	18.74	108.86	19.80	2449.00	26.85	82.18	2.23	0.19	Tropical monsoon forest	*Lauraceae, Fagaceae*	11	43

^†^HZ: Huzhong; LS: Liangshui; CB: Changbai; DL: Dongling; TY: Taiyue; SN: Shennong; JL: Jiulian; DH: Dinghu; JF: Jianfeng.

^‡^MAT: mean annual temperature; MAP: mean annual precipitation.

^§^Important values of all plant species were calculated according to relative density, relative frequency, and relative dominance^39^.

**Table 2:** Abbreviations, units, and description of morphological and anatomical leaf traits.

Leaf traits	Abbreviation	Units	Description
Morphological traits			
Leaf area	–¶	cm^2^ per individual	larger leaf area benefiting for light absorption
Leaf dry weight	–	mg per individual	Leaf construction investment index
Specific leaf area	SLA	mm^2^ mg^−1^	A comprehensive index reflecting plant photosynthetic capacity
Anatomical traits			
Stomatal length	–	μm	An index to describe stomata size
Stomatal density	–	individual mm^−2^	An index to describe stomata number
Stomatal pore index	SPI	%	An integrative parameter reflecting leaf stomatal conductance
Leaf thickness	–	μm	Leaf thickness index
Palisade-leaf mesophyll thickness ratio	–	%	Higher palisade mesophyll containing more chloroplasts and benefiting for light absorption
Spongy-leaf mesophyll thickness ratio	–	%	Higher spongy mesophyll benefiting for gas exchange inside leaf

^¶^“—” no abbreviations for the specific leaf trait.

**Table 3:** Changes in leaf morphological and anatomical traits among nine randomly selected forests along the north-south transect of eastern China.

Site	No. species	Morphological traits			Anatomical traits					
											Leaf area (cm^2^per individual)	Leaf dry weight (mg per individual)	Specific leaf area (mm^2^ mg^−1^)	Stomatal length (μm)	Stomatal density (individual mm^−2^)	Stomatal pore area index (%)	Leaf thickness (μm)	Palisade-leaf mesophyll thicknes ratio (%)	Spongy-leaf mesophyll thickness ratio(%)
HZ†	11	13.44 ± 3.68^a^	106.37 ± 36.82^ad^‡	15.41 ± 2.48^abd^	13.33 ± 1.01^abc^	238.88 ± 44.44^a^	3.80 ± 0.62^ab^	182.38 ± 36.95^a^	36.51 ± 2.94^ab^	39.86 ± 2.43^ab^
LS	11	26.12 ± 7.83^a^	111.11 ± 29.16^ad^	19.75 ± 4.30^a^	15.90 ± 2.42^a^	203.16 ± 39.70^a^	4.61 ± 1.01^ab^	123.32 ± 22.16^ab^	35.22 ± 2.28^ab^	34.39 ± 0.99^a^
CB	10	58.74 ± 14.70^b^	206.17 ± 54.65^abcd^	22.00 ± 2.29^a^	12.86 ± 1.23^abc^	321.51.7 ± 89.66^a^	4.16 ± 0.92^ab^	166.06 ± 21.03^a^	39.58 ± 3.01^a^	38.32 ± 2.47^ab^
DL	12	16.52 ± 4.35^a^	119.01 ± 34.89^ad^	15.39 ± 1.75^ab^	13.15 ± 1.09^abc^	220.58 ± 61.17^a^	3.01 ± 0.70^ab^	95.94 ± 13.71^bc^	36.65 ± 2.94^ab^	38.56 ± 3.28^ab^
TY	10	15.30 ± 3.78^a^	98.09 ± 13.90^a^	15.18 ± 2.31^abd^	14.44 ± 1.21^ac^	222.17 ± 58.15^a^	4.32 ± 1.10^ab^	77.58 ± 11.91^bc^	35.42 ± 3.11^ab^	43.63 ± 2.61^bc^
SN	13	17.19 ± 4.55^a^	135.09 ± 29.26^ad^	12.01 ± 2.56^bcde^	17.06 ± 2.33^a^	206.78 ± 43.93^a^	4.91 ± 1.35^a^	98.02 ± 11.19^b^	35.53 ± 2.44^ab^	42.96 ± 2.21^bc^
JL	10	20.59 ± 5.41^a^	268.43 ± 53.75^b^	7.87 ± 1.22^ce^	9.48 ± 1.34^b^	319.94 ± 46.85^a^	2.76 ± 0.76^ab^	129.42 ± 13.00^c^	30.53 ± 2.33^bc^	44.27 ± 2.43^bc^
DH	11	16.11 ± 2.77^a^	208.28 ± 31.38^bd^	8.68 ± 1.76^de^	9.01 ± 0.97^b^	335.66 ± 59.44^a^	2.38 ± 0.42^b^	160.36 ± 26.78^d^	30.10 ± 1.60^bc^	48.49 ± 2.56^cd^
JF	11	19.58 ± 5.59^a^	245.32 ± 36.26^bc^	7.69 ± 1.29^e^	10.73 ± 1.49^bc^	257.41 ± 53.70^a^	2.29 ± 0.44^b^	199.51 ± 32.80^d^	24.86 ± 2.08^c^	55.01 ± 3.37^d^
F		4.14	3.11	4.22	3.02	0.88	1.25	3.46	2.57	4.66
P		< 0.01	< 0.01	< 0.01	< 0.01	> 0.05	> 0.05	< 0.01	< 0.05	< 0.01

^†^HZ: Huzhong; LS: Liangshui; CB: Changbai; DL: Dongling; TY: Taiyue; SN: Shennong; JL: Jiulian; DH: Dinghu; JF: Jianfeng.

^‡^Data are presented as means ± standard errors. Different superscript letters indicated significant differences between forests at *P* < 0.05.

**Table 4:** Standardized effects of climate, soil nutrients, and leaf nutrients on morphological and anatomical traits.

		Factors	Direct effect
Morphological traits	Leaf area (cm^2^ per individual)	Climate†	**0.62**‡
		Soil nutrient	**0.65**
		Leaf nutrient	0.47
	Leaf dry weight (mg per individual)	Climate	0.04
		Soil nutrient	0.17
		Leaf nutrient	**0.78**
	Specific leaf area (mm^2^ mg^−1^)	Climate	**0.69**
		Soil nutrient	0.19
		Leaf nutrient	0.30
Anatomical traits	Stomatal length (μm)	Climate	**−0.52**
		Soil nutrient	0.06
		Leaf nutrient	−**0.50**
	Stomatal density (individual mm^−2^)	Climate	0.31
		Soil nutrient	0.21
		Leaf nutrient	0.04
	Stomatal pore area index (%)	Climate	−0.19
		Soil nutrient	0.19
		Leaf nutrient	−**0.22**
	Leaf thickness (μm)	Climate	0.45
		Soil nutrient	0.50
		Leaf nutrient	−0.45
	Palisade-leaf mesophyll thickness ratio (%)	Climate	−**0.51**
		Soil nutrient	0.17
		Leaf nutrient	−0.27
	Spongy-leaf mesophyll thickness ratio (%)	Climate	**0.26**
		Soil nutrient	−0.22
		Leaf nutrient	−0.12

^†^Climate refers to maximum monthly temperature and mean annual precipitation; Soil nutrients refer to soil organic carbon and soil total nitrogen; Leaf nutrients refer to leaf carbon and leaf nitrogen.

^‡^Bold numbers indicate environmental factors with a major effect on leaf traits.
