# Supplementary material for: Leaf morphological and anatomical traits from tropical to temperate coniferous forests: Mechanisms and influencing factors
Source: Sci Rep. 2016 Jan 22;6:19703. doi: 10.1038/srep19703 (PMC4726163; doi:10.1038/srep19703)
Supplement: Supplementary Information [file srep19703-s1.doc]

**Title:** Leaf morphological and anatomical traits from tropical to temperate coniferous forests: Mechanisms and controlling factors

**Authors**: Miao Tian ^1, 2^, Guirui Yu^1^, Nianpeng He ^1*^, Jihua Hou ^2*^

1 Key Laboratory of Ecosystem Network Observation and Modeling, Institute of Geographic Sciences and Natural Resources Research, Chinese Academy of Sciences, Beijing 100101, China

2 The Key Laboratory for Forest Resources & Ecosystem Processes of Beijing, Beijing Forestry University, Beijing 100083, China

**Corresponding author*. Nianpeng He and Jihua Hou

E-mail address: [henp@igsnrr.ac.cn](mailto:henp@igsnrr.ac.cn) (N.H.); [houjihua@bjfu.edu.cn](mailto:houjihua@bjfu.edu.cn) (J.H.)

**Table S1** Person’s correlations of leaf traits

|  | Leaf area  (cm^2^ individual^-1^) | Leaf dry weight  (mg individual^-1^) | Specific leaf area  (mm² mg^-1^) | Stomatal length  (μm) | Stomatal density  (individual mm^-2^) | Stomatal pore  area index (%) | Leaf thickness  (µm) | Palisade-leaf mesophyll thickness ratio (%) | Spongy-leaf mesophyll thickness ratio (%) |
| --- | --- | --- | --- | --- | --- | --- | --- | --- | --- |
| Leaf area | 1 |  |  |  |  |  |  |  |  |
| Leaf dry weight | 0.67^*** †^ | 1 |  |  |  |  |  |  |  |
| Specific leaf area | 0.29^**^ | -0.49^***^ | 1 |  |  |  |  |  |  |
| Stomatal length | 0.07 | -0.20^*^ | 0.36^***^ | 1 |  |  |  |  |  |
| Stomatal density | 0.05 | 0.32^**^ | -0.43^***^ | -0.43^***^ | 1 |  |  |  |  |
| Stomatal pore area index | 0.11 | 0.11 | -0.06 | 0.55^***^ | 0.52^***^ | 1 |  |  |  |
| Leaf thickness | 0.01 | 0.30^**^ | -0.43^***^ | -0.08 | 0.23^*^ | 0.14 | 1 |  |  |
| Palisade-leaf mesophyll thickness ratio | 0.08 | 0.01 | 0.07 | 0.04 | 0.40^***^ | 0.41^***^ | -0.14 | 1 |  |
| Spongy-leaf mesophyll thickness ratio | -0.20^*^ | 0.04 | -0.26^*^ | -0.25 | -0.25^*^ | -0.24^*^ | 0.24^*^ | -0.70^***^ | 1 |

^†^ *, *P* < 0.05; **, *P* < 0.01;***, *P* < 0.001.

Mean = 1.04; SE = 0.06

Max = 2.19; n = 98

Min = -1.39

Mean = 1.98; SE = 0.06

Max = 2.77; n = 98

Min = -0.39

Mean = 1.04; SE = 0.03

Max = 1.68; n = 98

Min = 0.28

Log specific leaf area (mm² mg^-1^)

Log leaf dry weight (mg individual^-1^)

Log leaf area (cm^2^ individual^-1^)

A

B

C

Mean = 1.08; SE = 0.02

Max = 1.56; n = 98

Min = 0.63

Log stomatal length (μm)

Mean = 2.28; SE = 0.04

Max = 2.92; n = 98

Min = 1.17

Log stomatal density (individual mm^-2^)

Mean = 0.44; SE = 0.03

Max = 1.29; n = 98

Min = -0.33

Log stomatal pore area index (%)

D

E

F

Mean = 2.07; SE = 0.02

Max = 2.71; n =99

Min = 1.60

Mean = 1.51; SE = 0.01

Max = 1.73; n = 83

Min = 1.16

Mean = 1.62; SE = 0.01

Max = 1.86; n = 83

Min = 1.32

G

H

I

Log palisade-leaf mesophyll thickness ratio (%)

Log spongy-leaf mesophyll thickness ratio (%)

Log leaf thickness (μm)

Frequency (%)

.

**Fig.S1** Frequency distribution of morphological (A, B, and C) and anatomical (D, E, F, G, H, and I) leaf traits.

A

R² = 0.65, *P* < 0.01

B

R² = 0.57, *P* < 0.05

C

R² = 0.77, *P* < 0.01

D

R² = 0.69, *P* < 0.01

ST/LT (%)

E

R² = 0.56, *P* < 0.05

Maximum monthly temperature **(°**C**)**

Maximum monthly temperature **(°**C**)**

Maximum monthly temperature **(°**C**)**

LD (mg individual^-1^)

SLA (mm² mg^-1^)

SPI (%)

PT/LT (%)

**Fig.S2** Changes in leaf morphological (A and B) and anatomical (C, D and E) traits with temperature. Bars represented standard errors. LD, leaf dry weight; SLA, specific leaf area; SPI, stomatal pore area index; PT/LT, palisade-leaf mesophyll thickness ratio; ST/LT, spongy-leaf mesophyll thickness ratio.

Stomatal density (individual mm^-2^)

Stomatal length (μm)

Palisade mesophyll thickness (μm)

Spongy mesophyll thickness (μm)

Anatomical traits

Fig. S3 Schematic for the measurement of leaf anatomical traits. Miao Tian took these pictures.

Stomatal image was photographed at 320× magnification using a scanning electron microscope (S-3400N, Hitachi, Japan), and leaf transection image was conducted at 400 × magnification with light microscope (Leica, DM2500, Germany).

ST (μm)

PT/ST (%)

PT (μm)

A

B

C

Latitude (**°**)

Latitude (**°**)

Latitude (**°**)

R² = 0.80, *P* < 0.01

R² = 0.80, *P* < 0.01

**Fig. S4** Latitudinal changes of leaf anatomical traits. Bars represented standard errors. PT, palisade mesophyll thickness; ST, spongy mesophyll thickness; PT/ST, palisade-spongy mesophyll thickness ratio.
